# Autologous fecal microbiota transplantation restores the infant gut microbiome and metabolome after antibiotics: a case report

**DOI:** 10.1128/mbio.00711-26

**Published:** 2026-05-29

**Authors:** Haipeng Sun, Anna Dulencin, Thomas J. Kirn, Jasmine Vo, Ivan Liachko, Deeptha Rao, Joseph Manzano-Santana, Ekta Patel, Connie Looi, Daniel B. Horton, Emily Barrett, Melissa Weidner, Gloria Bachmann, Reynold A. Panettieri, Bradley A. Connor, Marina Rogova, Dorottya Nagy-Szakal, Mara Couto-Rodriguez, Shashikant Kotwal, Qingli Wu, James Simon, Martin J. Blaser, Maria Gloria Dominguez Bello

**Affiliations:** 1 Department of Biochemistry and Microbiology, Rutgers University, New Brunswick, New Jersey, USA; 2 Department of Pathology and Laboratory Medicine, Rutgers Robert Wood Johnson Medical School 12287 , New Brunswick, New Jersey, USA; 3 Department of Pharmacology and Toxicology, Ernest Mario School of Pharmacy, Rutgers University New Brunswick, Piscataway, New Jersey, USA; 4 Phase Genomics 581436 , Seattle, Washington, USA; 5 Department of Pediatrics, Rutgers Robert Wood Johnson Medical School 12287 , New Brunswick, New Jersey, USA; 6 Rutgers Center for Pharmacoepidemiology and Treatment Science, Healthcare Policy and Aging Research, Institute for Health, New Brunswick, New Jersey, USA; 7 Department of Biostatistics and Epidemiology, Rutgers School of Public Health 51893 , Piscataway, New Jersey, USA; 8 Department of Obstetrics, Gynecology, and Reproductive Sciences, Rutgers Robert Wood Johnson Medical School 12287 , New Brunswick, New Jersey, USA; 9 Rutgers Institute for Translational Medicine and Science 827782 , New Brunswick, New Jersey, USA; 10 Department of Medicine, Rutgers Robert Wood Johnson Medical School 12287 , New Brunswick, New Jersey, USA; 11 The New York Center for Travel and Tropical Medicine, Weill Cornell Medical College 12295 , New York, New York, USA; 12 Biotia Laboratory, Queens, New York, USA; 13 Department of Cell Biology, College of Medicine, SUNY Downstate Health Sciences University 12298, Brooklyn, New York, USA; 14 Department of Plant Biology, Rutgers University New Brunswick, New Brunswick, New Jersey, USA; 15 Center for Advanced Biotechnology and Medicine, Rutgers University New Brunswick 5970, Piscataway, New Jersey, USA; 16 Humans and the Microbiome Program, Canadian Institute for Advanced Research (CIFAR), Toronto, Ontario, Canada; 17 Department of Anthropology, Rutgers University New Brunswick 5970, New Brunswick, New Jersey, USA; Universiteit Gent, Gent, Belgium

**Keywords:** infant microbiome, antibiotic perturbation, fecal microbiota transplantation

## Abstract

Antibiotic exposure during infancy disrupts gut microbiome assembly during a critical developmental window. Strategies to restore these ecosystems remain limited. In the REPAIR trial (NCT06609980), eight infants were followed longitudinally; two received amoxicillin for otitis media, and one subsequently underwent autologous fecal microbiota transplantation (aFMT) using stool collected prior to antibiotic exposure. Shotgun metagenomics, Hi-C–assisted resistome profiling, and untargeted metabolomics were performed on samples collected before and after antibiotics. Amoxicillin treatment was associated with displacement of community structure, enrichment of antibiotic resistance genes (ARGs), and altered fecal metabolites, including short-chain fatty acids, bile acids, acylcarnitines, bilirubin derivatives, tricarboxylic acid (TCA) cycle metabolites, and amino acids. In the non-restored infant, microbiota composition and ARG profiles remained persistently altered during follow-up, accompanied by sustained metabolic divergence. In contrast, the aFMT-treated infant demonstrated convergence toward pre-antibiotic community structure, directional restructuring of ARG carriers —including reduction of β-lactam and tetracycline resistance genes— and metabolite profiles trending toward the pre-antibiotic baseline across analytical platforms. Although limited to a case-based comparison, these findings provide integrated ecological and functional evidence that aFMT may promote recovery following antibiotic perturbation during early-life microbiome development and support the rationale for larger controlled clinical trials.

**IMPORTANCE:**

Antibiotic exposure in early life disrupts the developing gut microbiome during a critical window of host-microbe interaction. However, the extent to which these disturbances resolve naturally, or can be actively reversed, remains unclear. In this study, we use longitudinal sampling in infants to examine microbiome recovery following antibiotics, with and without autologous fecal microbiota transplantation (aFMT). We show that antibiotic exposure leads to coordinated disruptions in microbial composition, antibiotic resistance genes, and metabolic profiles. While partial recovery spontaneously occurs over time, faster and more extensive restoration toward the pre-antibiotic state is observed following aFMT. These findings provide insight into the ecological dynamics of microbiome reassembly in early life and highlight the potential of using controlled perturbations to understand microbiome resilience.

**CLINICAL TRIALS:**

This study is registered with ClinicalTrials.gov as NCT06609980.

## OBSERVATION

Antibiotics are frequently prescribed during infancy and disrupt the developing gut microbiota at a critical window of host–microbiome co-maturation (1, 2). Early-life perturbations have been associated with increased risks of immune and metabolic disorders later in childhood (3–7). Recovery following antibiotic exposure is often incomplete (8, 9), and treatment can promote enrichment of antimicrobial resistance genes (ARGs) within the gut microbiome (10). Strategies to restore the native microbiota after antibiotic exposure remain limited.

Fecal microbiota transplantation (FMT) restores intestinal microbial communities in adults, notably for recurrent *Clostridioides difficile* infection (11, 12). However, its use during early-life microbiome assembly remains limited due to concerns regarding pathogen transmission and long-term safety. Autologous fecal microbiota transplantation (aFMT), which uses stool collected from the same individual prior to antibiotic exposure, mitigates donor-related risks and represents a biologically targeted strategy to promote ecological recovery. Experimental and adult clinical studies suggest that aFMT can facilitate microbiome reconstitution following antibiotic perturbation (13–16) although its impact during infancy has not been characterized.

**Fig 1:**
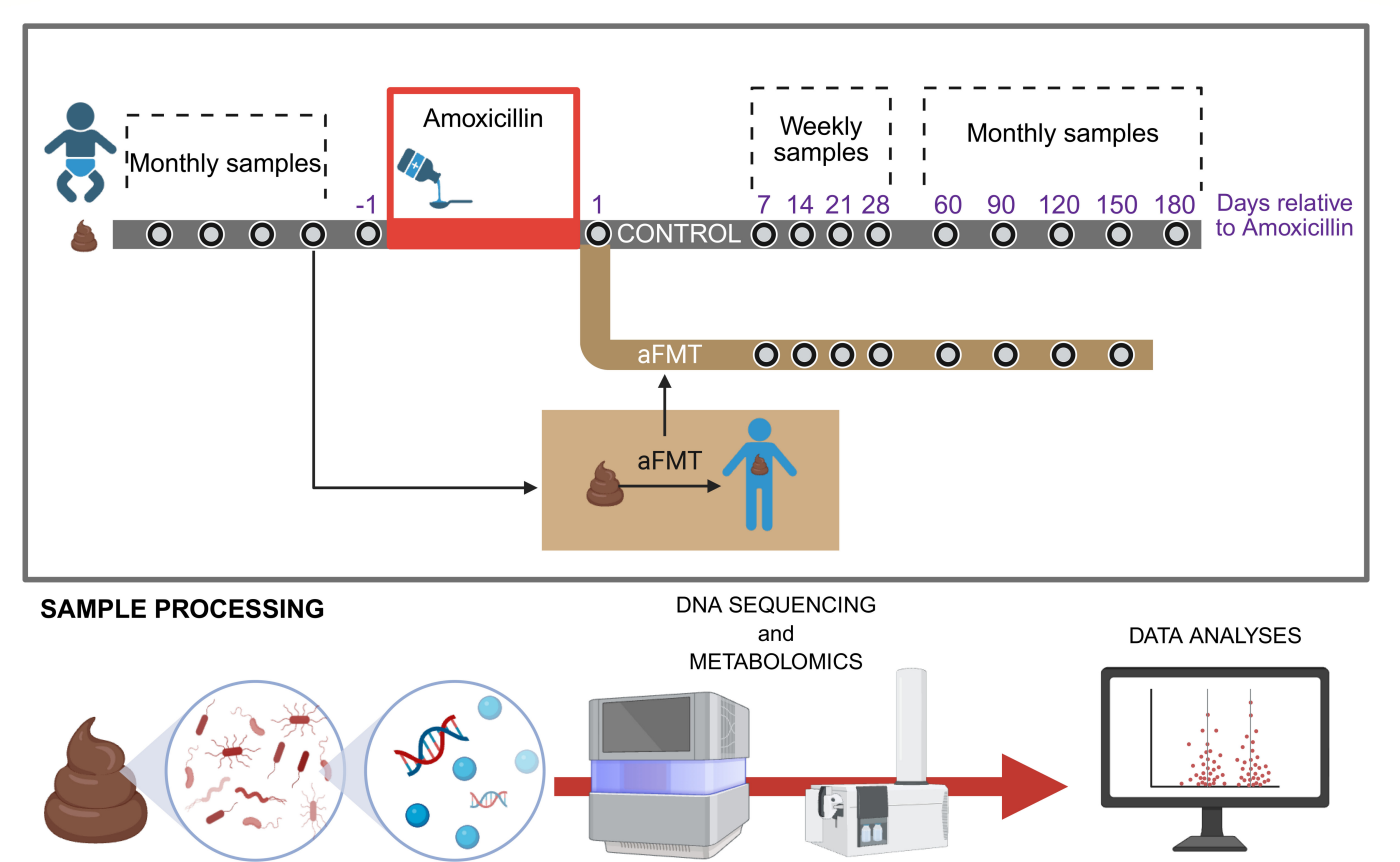
Study design and sample processing workflow. Two children were followed longitudinally with monthly stool sampling prior to antibiotic exposure. The day before (−1) and after (+1) the antibiotic course, additional samples were collected, followed by weekly sampling and subsequent monthly sampling through the end of a 6-month follow-up. One child received an autologous fecal microbiota transplant (aFMT) after completion of an antibiotic course (Baby 2; Restored), but the other (Baby 1; Control) did not. Samples were processed for DNA metagenomics and metabolomics. Figure created in BioRender.

In the REPAIR trial (NCT06609980), eight infants were followed longitudinally with monthly fecal sampling during the first year of life (Fig. 1; Table S1). Two received a 10-day course of amoxicillin for otitis media as part of routine clinical care. One of these infants subsequently underwent aFMT using stool collected prior to antibiotic exposure, whereas the other did not receive restoration. Here, we compare longitudinal microbiome structure, resistome dynamics, and fecal metabolomic profiles in these two antibiotic-exposed infants—one restored and one not restored—within the context of six untreated reference children. To our knowledge, this is among the first longitudinal human studies to directly compare post-antibiotic microbiome recovery trajectories with and without autologous restoration in early life.

### Microbiota composition and ecological recovery

**Fig 2:**
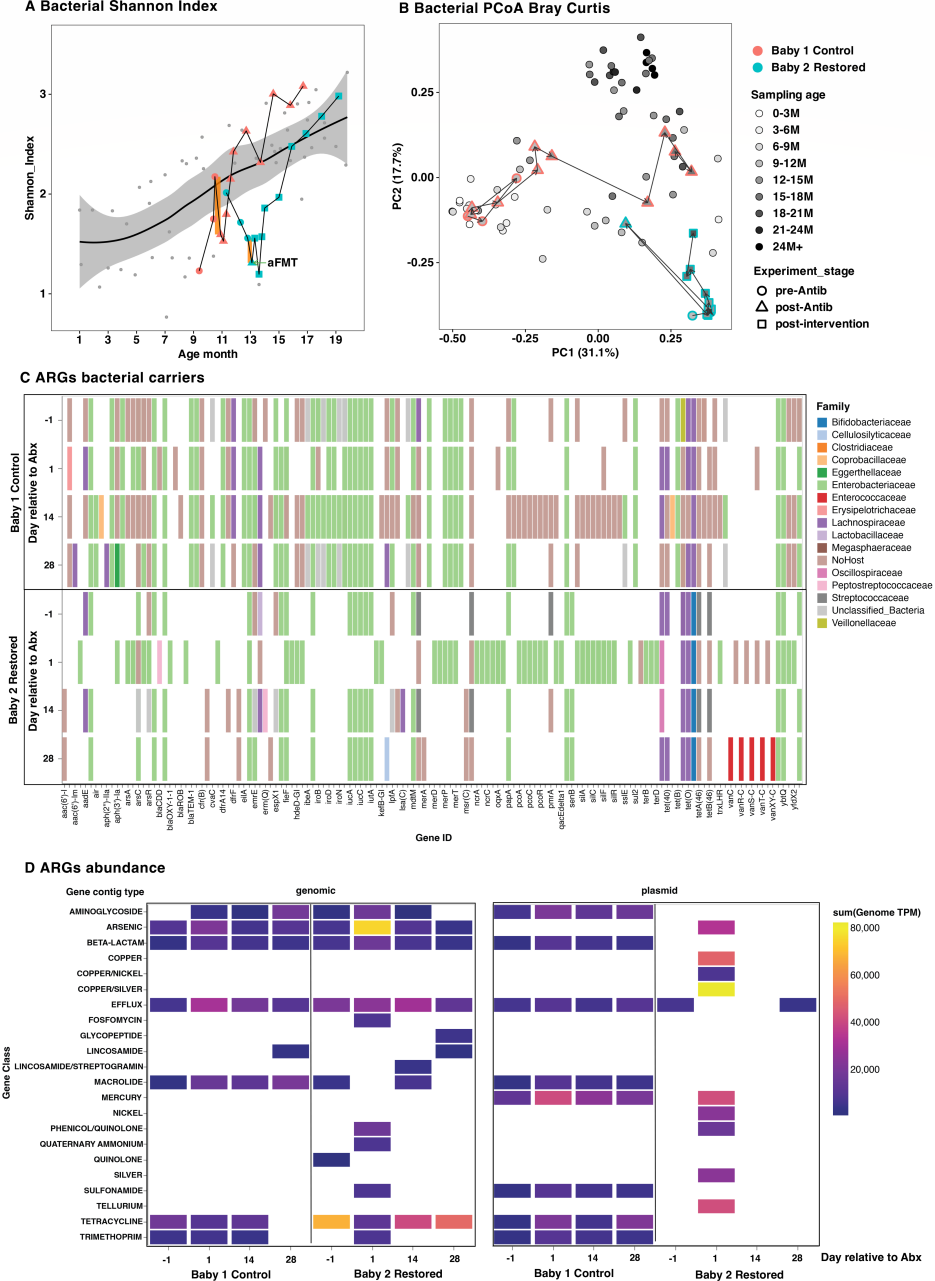
Microbiota β-diversity and antibiotic resistance gene (ARG) profiles before and after antibiotic exposure and aFMT. (**A**) Shannon diversity by sampling age. Gray dots indicate healthy reference samples, with a black line and shaded area indicating the mean and 95% confidence interval. Baby 1 Control in red and Baby 2 Restored in green; the circle indicates pre-antibiotic samples, the triangle indicates post-antibiotic samples, and the square indicates post-intervention samples. The antibiotic treatment periods were indicated in orange squares. (**B**) Principal coordinates analyses (PCoA) of Bray–Curtis distances. Gray dots indicate healthy reference samples, gradient by age. Baby 1 Control in red and Baby 2 Restored in green; the circle indicates pre-antibiotic samples, the triangle indicates post-antibiotic samples, and the square indicates post-intervention samples. (**C**) Bacterial families carrying ARGs across sampling time points for Baby 1 (Control) and Baby 2 (Restored). Each vertical bar represents the presence of an ARG within a contig assigned to a given family. (**D**) Abundance of ARG classes detected on genomic and plasmid contigs in Baby 1 (Control) and Baby 2 (Restored). Values reflect the summed genome-normalized transcript per million (TPM) for each ARG class at each time point.

Alpha diversity (Shannon index) declined transiently following antibiotic exposure in both infants and recovered toward baseline after about 14 days (Fig. 2A).

Amoxicillin exposure was associated with marked shifts in gut microbial community structure in both infants. In the non-restored infant (Baby 1), Bray–Curtis β-diversity analysis demonstrated sustained displacement from the pre-antibiotic baseline that persisted across follow-up. In contrast, the restored infant (Baby 2) showed convergence toward the pre-antibiotic configuration within 14 days following aFMT (Fig. 2B).

Across the broader cohort (*n* = 8), permutational multivariate analysis of variance indicated that experimental stage significantly contributed to variation in microbial composition (Table S2), supporting the interpretation that antibiotic exposure represented a major ecological perturbation.

Taxonomic responses differed between the two infants (Fig. S1 and S2). The restored infant demonstrated recovery of *Bifidobacterium* and reduced persistence of facultative anaerobes relative to the non-restored infant.

### Antibiotic resistance gene dynamics

Patterns of antibiotic resistance gene (ARG) carriage differed between the two antibiotic-exposed infants (Fig. 2C and D). The non-restored infant (Baby 1) exhibited a higher baseline burden of ARG-associated contigs prior to antibiotic exposure. At baseline, ARGs included β-lactam resistance genes (*blaTEM-1* and *blaSHV*), tetracycline resistance genes (*tetM*, *tetO*, and *tetW*), macrolide resistance genes (*ermB* and *mefA*), aminoglycoside resistance genes (*aac*, *aph*, and *aadA*), and sulfonamide resistance genes (*sul1* and *sul2*). These determinants were detected on both chromosomal and plasmid-associated contigs and were primarily carried by members of the Enterobacteriaceae, Enterococcaceae, and Lachnospiraceae families (Fig. 2C and D).

Following antibiotic exposure, ARG profiles in Baby 1 remained relatively stable across post-antibiotic time points, with persistent representation of β-lactam and tetracycline resistance genes (Fig. 2D; Table S3). In contrast, antibiotic exposure in the restored infant (Baby 2) was associated with transient enrichment of ARGs within Enterobacteriaceae and Lachnospiraceae (Fig. 2C). After autologous fecal microbiota transplantation (aFMT), the relative contribution of these ARG carriers declined.

Ordination of ARG profiles showed that Baby 2 deviated most strongly from baseline at day 1 after antibiotic exposure but returned toward the pre-antibiotic configuration by day 14, whereas Baby 1 remained relatively stable across time points (Fig. S3). Consistent with these patterns, ARG class abundance analysis revealed persistent enrichment of β-lactam and tetracycline resistance genes in Baby 1, whereas these classes declined following aFMT in Baby 2 (Fig. 2D). Given the limited sample size, these comparisons are interpreted descriptively, and formal statistical testing of ARG reversion was not powered to detect differences between individuals.

### Metabolomic shifts following antibiotic exposure and aFMT

**Fig 3:**
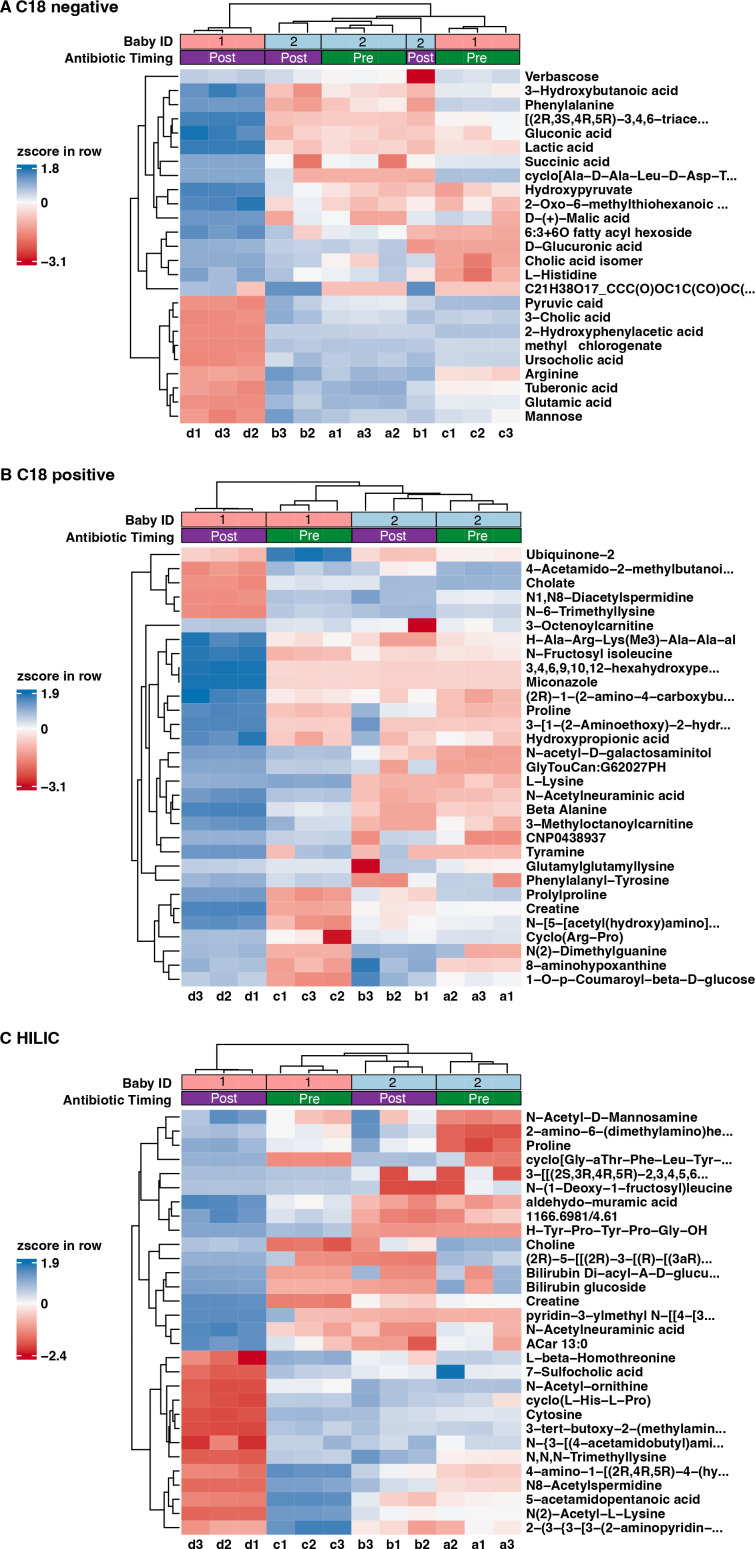
Metabolite profiles 1 day before and 7 days after antibiotic exposure in the control and restored babies. Metabolites detected in C18 negative mode (**A**)**,** C18 positive mode (**B**), and HILIC mode (**C**) for Baby 1 and Baby 2 at pre-antibiotic and post-antibiotic time points. Showing metabolites that are significantly different between pre-antibiotic (of both babies) and post-antibiotic Baby 1, but not post-antibiotic Baby 2. Statistical test was performed using MetaboAnalyst 6.0. Rows represent metabolites, and columns show three biological replicates per sample. Values are displayed as row-wise *Z* scores. Only annotated metabolites were shown.

Metabolites were detected using complementary chromatographic and ionization methods used in liquid chromatography–mass spectrometry (LC–MS), namely, using C18 negative, C18 positive, and hydrophilic interaction liquid chromatography (HILIC) platforms. Hierarchical clustering of metabolomic profiles across these platforms revealed clear shifts in metabolite composition following antibiotic exposure (Fig. 3A through C).

The non-restored infant (Baby 1) showed persistent separation between pre- and post-antibiotic samples, indicating sustained fecal metabolome disruption. In contrast, the restored infant (Baby 2) exhibited partial convergence toward pre-antibiotic metabolome profiles following aFMT (Fig. 3A through C).

Untargeted metabolomics revealed that antibiotic exposure was associated with decreased primary bile acid derivatives, including 7-sulfocholic acid, 3-cholic acid, ursocholic acid, as well as metabolites related to short-chain fatty acids such as 3-hydroxybutanoic acid, hydroxypropionic acid, and 4-acetamido-2-methylbutanoic acid (17, 18).

Conversely, antibiotic exposure was associated with increased levels of acylcarnitines, bilirubin derivatives, sialic acid, and tricarboxylic acid cycle-related metabolites, which include 3-methyloctanoylcarnitine, 3-octenoylcarnitine, bilirubin glucosides, N-acetylneuraminic acid, and succinic acid, consistent with metabolomic patterns associated with gut dysbiosis in Baby 1 at day 7 post-antibiotic treatment (19–22).

In addition, several amino acids and small peptides, including creatine, L-histidine, proline, L-lysine, and β-alanine, accumulated after post-antibiotic exposure. In Baby 2, the abundance of these metabolites shifted toward pre-antibiotic state following aFMT, consistent with recovery of the fecal metabolome (Fig. 3A through C).

### Interpretation

This case-based comparison provides integrated ecological and functional evidence that autologous fecal microbiota transplantation (aFMT) may promote recovery of the infant gut microbiome following antibiotic perturbation. In the restored infant, aFMT was associated with convergence of microbial community composition toward the pre-antibiotic state, reduction of antibiotic resistance genes that increased following antibiotic exposure, and partial restoration of altered fecal metabolite profiles. In contrast, the non-restored infant showed persistent divergence in microbiota composition, resistome structure, and metabolomic profiles during follow-up.

Notably, the two infants differed in their baseline abundance of antibiotic resistance genes despite neither having received antibiotics prior to the study, highlighting the variability of early-life resistome composition and the potential influence of other clinical or environmental exposures.

Together, these findings suggest that early-life antibiotic exposure induces a reproducible ecological disturbance but that full recovery across taxonomic, genetic, and functional dimensions may not occur spontaneously. Instead, targeted restoration via aFMT appears capable of redirecting microbiome assembly toward its pre-perturbation trajectory during a critical developmental window.

Given the case-based design, these observations should be interpreted with caution, and larger cohorts will be required to disentangle restoration effects from inter-individual variability.

Infants were enrolled and followed longitudinally, with stool samples collected monthly and stored at −80°C for downstream analyses. When clinically indicated, antibiotic exposure occurred as part of routine pediatric care and was not dictated by the study protocol.

Infants whose parents consent to participation in the intervention group undergo autologous fecal microbiota transplantation (aFMT). For aFMT, the stool collected from the same infant prior to antibiotic treatment is used as the source material. The stool is screened for potential gastrointestinal pathogens and confirmed negative before preparation. A standardized stool suspension is then prepared and administered orally by bottle on the day following completion of the antibiotic course, after collection of the first post-antibiotic stool sample.

Participant characteristics for the first eight children enrolled in the study are summarized in Table S1. Two children were prescribed a 10-day course of amoxicillin by their pediatrician for otitis media and underwent additional sampling 1 day before antibiotic initiation (day −1), 1 day after completion of antibiotics (day 1), weekly during the first month, and monthly thereafter through 6 months. For one of the antibiotic-exposed children whose parents consented to the intervention, autologous fecal microbiota transplantation (aFMT) was performed using stool collected prior to symptom onset and 14 days before antibiotic exposure. The fecal sample screen targets are listed in Tables S4 and S5. The stool suspension was administered by bottle after collection of the first post-antibiotic stool sample, on the day following completion of the antibiotic course (day 1).

Fecal DNA from the eight children was profiled by 16S rRNA amplicon sequencing targeting V4 hypervariable region (Primers 515 F 5′-GTGYCAGCMGCCGCGGTAA-3′ and 806R 5′-GGACTACNVGGGTWTCTAAT-3′) following protocols from the Earth Microbiome Project (23). Amplicons were sequenced on an Illumina MiSeq platform. Raw reads were demultiplexed and quality-filtered using QIIME2 pipeline (24) and denoised with DADA2 (25). Taxonomy was assigned to Amplicon sequence variants (ASVs) using Greengene2 (version 2024.9) as the reference database (26).

Fecal samples collected 1 day before the antibiotic course (day −1) and on days 1, 14, and 28 after completion of antibiotics were submitted to Phase Genomics (Seattle, WA, USA) for shotgun metagenomic and Hi-C sequencing using a proximity ligation (Hi-C) kit (18). Sequencing data were processed and visualized using the Phase Genomics cloud-based bioinformatics platform, ProxiMeta Explorer.

Fecal samples collected 1 day before the antibiotic course and on day 7 after completion of antibiotics from the two children were analyzed by untargeted metabolomics. Sample preparation for chemical analysis followed protocols similar to those described by Han et al. (27). Untargeted metabolomics was performed using an Agilent 1290 Infinity II UHPLC system coupled to an Agilent 6546 quadrupole time-of-flight mass spectrometer (UHPLC–QTOF/MS), operated in both positive (ESI^+^) and negative (ESI⁻) electrospray ionization modes with complementary chromatographic approaches to maximize metabolite coverage.

C18 Positive Mode (reverse-phase LC–MS, positive ionization) was used to detect moderately polar or hydrophobic metabolites that ionize efficiently by protonation, including lipids, fatty acids, bile acids, steroids, and selected amino acids and phenolic compounds. C18 Negative Mode (reverse-phase LC–MS, negative ionization) was used to detect moderately polar or acidic metabolites that ionize efficiently by deprotonation, including organic acids, fatty acids, bile acids, phospholipids, and microbial metabolites. Hydrophilic interaction liquid chromatography (HILIC) was used to detect highly polar metabolites, including sugars, amino acids, nucleotides, and central carbon metabolism intermediates. Mobile-phase solvents and chromatographic gradients followed protocols similar to those described by Anderson et al. (28), with minor modifications.

Peak areas of molecular features were extracted using Agilent MassHunter Profinder (version 10.0), filtered using R (version 4.4.2), and imported into MetaboAnalyst (version 6.0) for data normalization and statistical analysis (29). MassHunter Qualitative Analysis (version 12.0), MS-DIAL, MS-CleanR, MS-FINDER, MetaboKit, and SIRIUS software, together with corresponding mass spectral libraries, were used for MSI level 2 compound identification (30–35). For visualization, data were normalized and transformed to row-wise Z scores.

## Data Availability

*16S rRNA* gene sequencing data have been deposited in the NCBI Sequence Read Archive under accession number PRJNA1347774. Shotgun metagenomic sequencing data are available in the European Nucleotide Archive under accession number PRJEB80456.

## SUPPLEMENTAL MATERIAL

Figure S1 mbio.00711-26-s0001.tif Relative abundance of the top antibiotic-responsive ASVs in the control and restored babies.

Figure S2 mbio.00711-26-s0002.tif Representative antibiotic-responsive ASV read counts in the control and restored babies.

Figure S3 mbio.00711-26-s0003.tif PCoA based on ARG profiles.

Supplemental Legends mbio.00711-26-s0004.docx Legends for supplemental figures and tables.

Supplemental Tables mbio.00711-26-s0005.docx Tables S1 to S5.

ASM does not own the copyrights to Supplemental Material that may be linked to, or accessed through, an article. The authors have granted ASM a non-exclusive, world-wide license to publish the Supplemental Material files. Please contact the corresponding author directly for reuse.
