# Supplementary figures and images for: Autologous fecal microbiota transplantation restores the infant gut microbiome and metabolome after antibiotics: a case report
Source: mBio. 2026 May 29;17(7):e00711-26. doi: 10.1128/mbio.00711-26 (PMC13343926; doi:10.1128/mbio.00711-26)

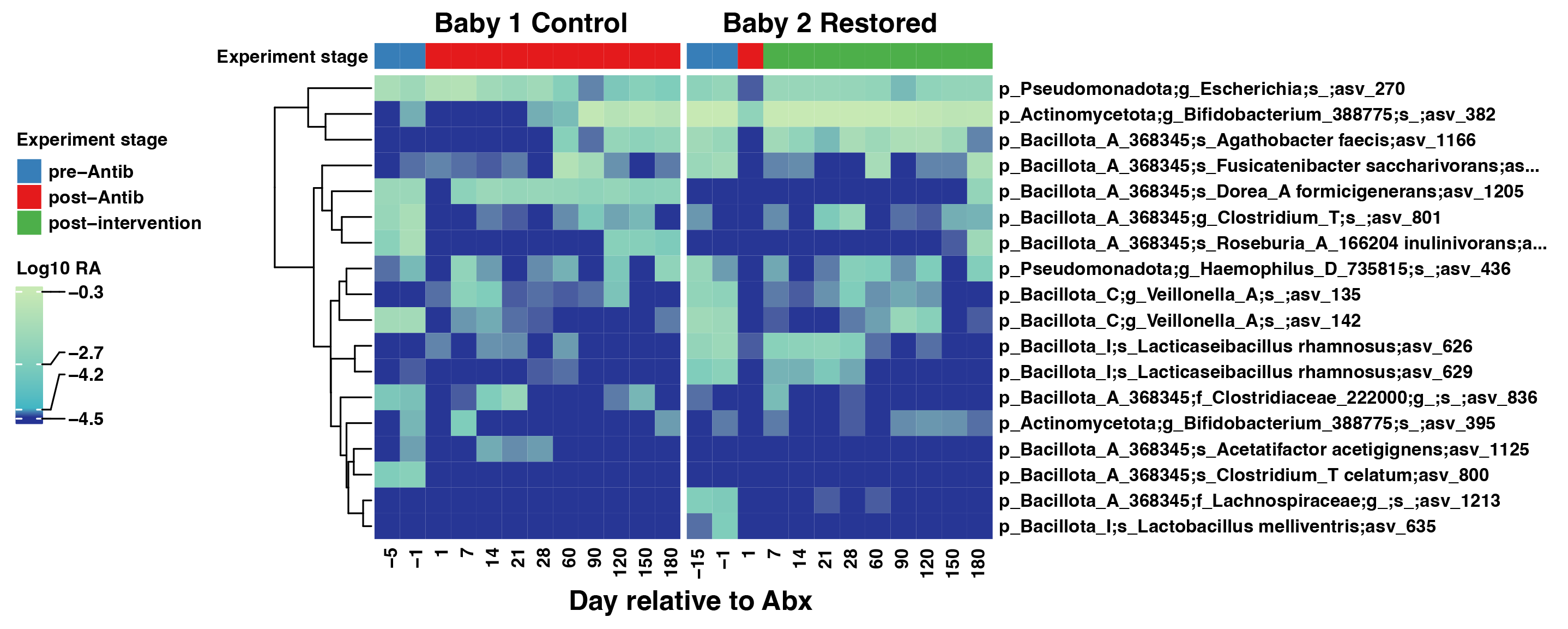

Supplement: Figure S1 — Relative abundance of the top antibiotic-responsive ASVs in the control and restored babies. [file mbio.00711-26-s0001.tif]

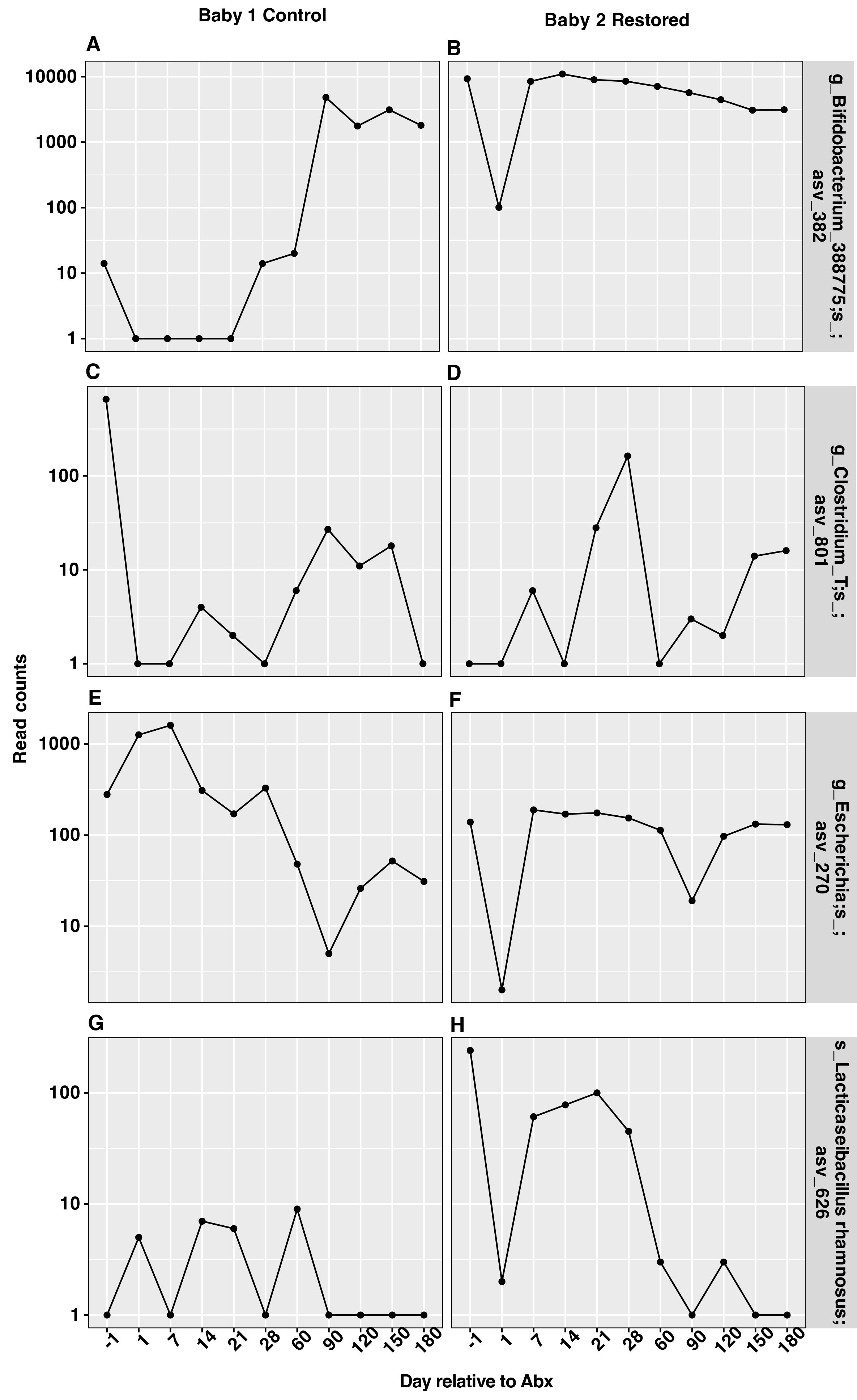

Supplement: Figure S2 — Representative antibiotic-responsive ASV read counts in the control and restored babies. [file mbio.00711-26-s0002.tif]

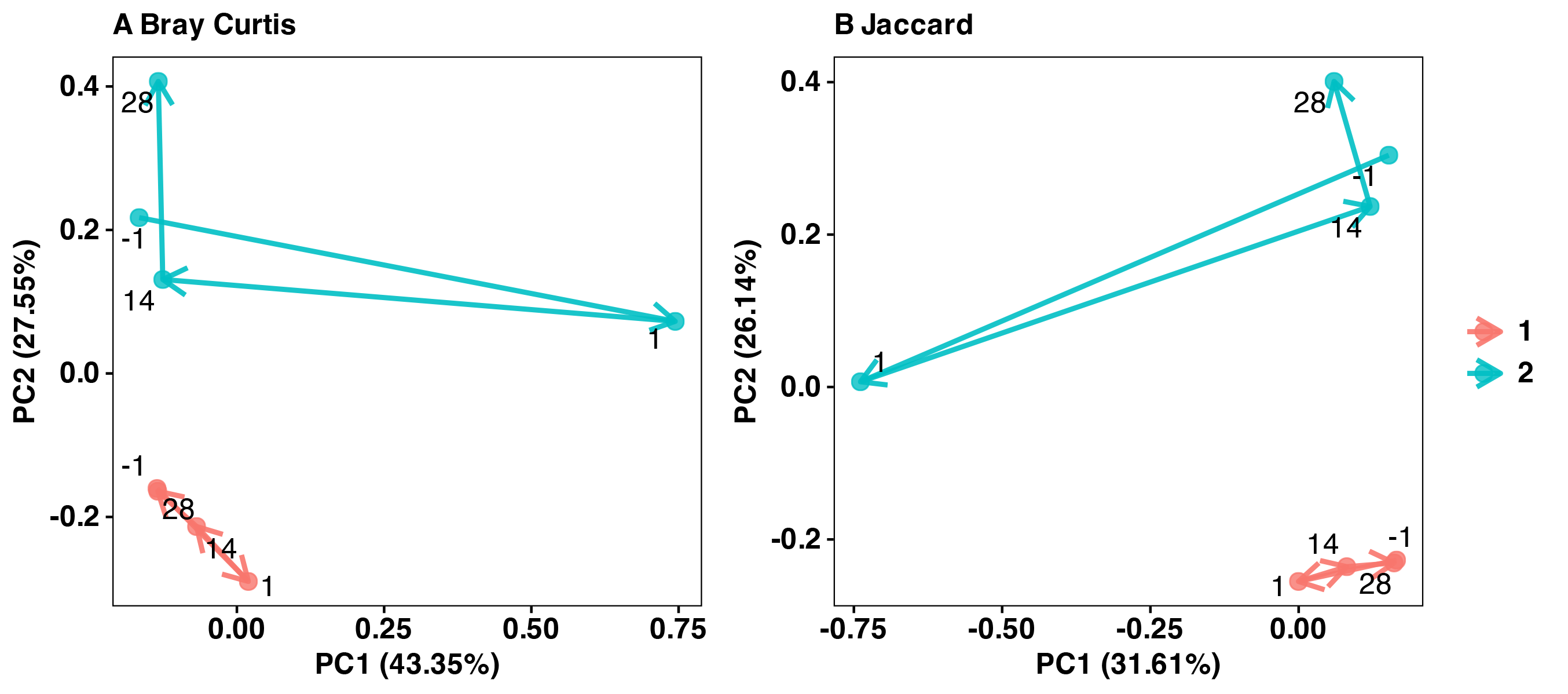

Supplement: Figure S3 — PCoA based on ARG profiles. [file mbio.00711-26-s0003.tif]
