# Supplementary material for: Autologous fecal microbiota transplantation restores the infant gut microbiome and metabolome after antibiotics: a case report
Source: mBio. 2026 May 29;17(7):e00711-26. doi: 10.1128/mbio.00711-26 (PMC13343926; doi:10.1128/mbio.00711-26)
Supplement: Supplemental Legends — Legends for supplemental figures and tables. [file mbio.00711-26-s0004.docx]

**Fig. S1. Relative abundance of the top antibiotic-responsive ASVs in the control and restored babies.**

Timepoint is in days relative to antibiotic treatment (e.g. pre-antibiotic day −1, and post-antibiotic (day 1, 7, 14, 21, 28, etc).

**Fig. S2. Representative antibiotic-responsive ASV read counts in the control and restored babies.**

Line plots showing read counts over time for selected ASVs in Baby 1 (Control) and Baby 2 (Restored). Ages at sampling were 10 months (Baby 1) and 12 months (Baby 2).

**(A–B)** Read counts for *Bifidobacterium* sp. (ASV_382) in Baby 1 and Baby 2.
**(C–D)** Read counts for *Clostridium* sp. (ASV_801) in Baby 1 and Baby 2.
**(E–F)** Read counts for *Escherichia* sp. (ASV_270) in Baby 1 and Baby 2.
**(G–H)** Read counts for *Lacticaseibacillus rhamnosus* (ASV_626) in Baby 1 and Baby 2.

**Fig. S3. Principal coordinates analyses (PCoA) based on ARG profiles**.

(A) Bray Curtis distance based PCoA, (B) Jaccard distance based PCoA.

**Table S1. Characteristics of children who received antibiotics and reference children.**

**Table S2. PERMANOVA results for beta diversity in all children (n = 8) across ages 1–20 months.**

PERMANOVA results based on Bray–Curtis and Jaccard distances testing the effects of age, birth mode, experiment stage, and sex on community composition. Experimental stages include three categories: pre-antibiotic, post-antibiotic, and post-intervention (restored).

**Table S3. Fisher’s exact test results of the types of detected ARGs.**

Pairwise Fisher’s exact test between different time points and babies for the number of different ARGs detected. P-value was adjusted by FDR.

**Table S4. Gastrointestinal pathogens to be screened in donor feces using the BioFire kit**

**Table S5. Additional gastrointestinal pathogens to be screened in donor feces.**
