# Supplementary material for: Autologous fecal microbiota transplantation restores the infant gut microbiome and metabolome after antibiotics: a case report
Source: mBio. 2026 May 29;17(7):e00711-26. doi: 10.1128/mbio.00711-26 (PMC13343926; doi:10.1128/mbio.00711-26)
Supplement: Supplemental Tables — Tables S1 to S5. [file mbio.00711-26-s0005.docx]

**Table S1. Characteristics of infants who received antibiotics and reference infants.**

| **Baby ID** | **Baby 1 control** | **Baby 2 restored** | **Baby 3** | **Baby 4** | **Baby 6** | **Baby 11** | **Baby 12** | **Baby 14** |
| --- | --- | --- | --- | --- | --- | --- | --- | --- |
| Baby group | Antibiotics | Antibiotics + aFMT | Healthy  reference | Healthy  reference | Healthy  reference | Healthy  reference | Healthy  reference | Healthy  reference |
| Sampling age range (months) | 9 - 17 | 11 - 19 | 14 - 20 | 14 - 20 | 7 - 19 | 1 -17 | 1- 10 | 6 - 10 |
| Age at antibiotics consumption (months) | 10 | 12 | _ | _ | _ | _ | _ | _ |
| Amoxicillin Dose | 400 mg/5 mL every 12 h | 400 mg/5 mL every 12 h | _ | _ | _ | _ | _ | _ |
| Amoxicillin course duration (days) | 10 | 10 | _ | _ | _ | _ | _ | _ |
| Birth mode | Vaginal | C-section | Vaginal | Vaginal | Vaginal | C-section | Vaginal | Vaginal |
| Sex | M | F | F | F | M | M | M | M |

**Table S2. PERMANOVA results for beta diversity in all infants (n = 8) across ages 1–20 months.**

PERMANOVA results based on Bray–Curtis and Jaccard distances testing the effects of age, birth mode, experiment stage, and sex on community composition. Experimental stages include three categories: pre-antibiotic, post-antibiotic, and post-intervention (restored).

|  | **Variable** | **Df** | **R2** | **Omega2_partial** | **F** | **Pr(>F)** |
| --- | --- | --- | --- | --- | --- | --- |
| Bray | Age | 1 | 0.129 | 0.159 | 17.436 | 0.001 |
|  | Birth_mode | 1 | 0.055 | 0.068 | 7.396 | 0.001 |
|  | Experiment_stage | 2 | 0.066 | 0.074 | 4.488 | 0.001 |
|  | Sex | 1 | 0.056 | 0.071 | 7.637 | 0.001 |
| Jaccard | Age | 1 | 0.093 | 0.102 | 10.917 | 0.001 |
|  | Birth_mode | 1 | 0.042 | 0.043 | 4.937 | 0.001 |
|  | Experiment_stage | 2 | 0.058 | 0.052 | 3.395 | 0.001 |
|  | Sex | 1 | 0.046 | 0.048 | 5.377 | 0.001 |

**Table S3. Fisher’s exact test results of the types of detected ARGs.**

Pairwise Fisher’s exact test between different time points and babies for the number of different ARGs detected. P-value was adjusted by FDR. The red cells show statistically significant differences in the number of antibiotic resistance genes (ARGs) detected between the two compared groups, after correction for multiple testing.

| Group1 | Group2 | N ARG in Group1 | N ARG in Group2 | p | p.adj |
| --- | --- | --- | --- | --- | --- |
| Baby_1_day_-1 | Baby_1_day_1 | 75 | 66 | 0.339 | 1.000 |
| Baby_1_day_-1 | Baby_1_day_14 | 75 | 99 | 0.004 | 0.054 |
| Baby_1_day_-1 | Baby_1_day_28 | 75 | 73 | 0.905 | 1.000 |
| Baby_1_day_1 | Baby_1_day_14 | 66 | 99 | 0.000 | 0.002 |
| Baby_1_day_1 | Baby_1_day_28 | 66 | 73 | 0.473 | 1.000 |
| Baby_1_day_14 | Baby_1_day_28 | 99 | 73 | 0.002 | 0.031 |
| Baby_2_day_-1 | Baby_2_day_1 | 36 | 68 | 0.000 | 0.002 |
| Baby_2_day_-1 | Baby_2_day_14 | 36 | 43 | 0.426 | 1.000 |
| Baby_2_day_-1 | Baby_2_day_28 | 36 | 42 | 0.505 | 1.000 |
| Baby_2_day_1 | Baby_2_day_14 | 68 | 43 | 0.003 | 0.043 |
| Baby_2_day_1 | Baby_2_day_28 | 68 | 42 | 0.002 | 0.031 |
| Baby_2_day_14 | Baby_2_day_28 | 43 | 42 | 1.000 | 1.000 |
| Baby_1_day_-1 | Baby_2_day_-1 | 75 | 36 | 0.000 | 0.000 |
| Baby_1_day_1 | Baby_2_day_1 | 66 | 68 | 0.905 | 1.000 |
| Baby_1_day_14 | Baby_2_day_14 | 99 | 43 | 0.000 | 0.000 |
| Baby_1_day_28 | Baby_2_day_28 | 73 | 42 | 0.000 | 0.005 |
| Baby_1_day_-1 | Baby_2_day_1 | 75 | 68 | 0.473 | 1.000 |
| Baby_1_day_-1 | Baby_2_day_14 | 75 | 43 | 0.000 | 0.003 |
| Baby_1_day_-1 | Baby_2_day_28 | 75 | 42 | 0.000 | 0.002 |
| Baby_1_day_1 | Baby_2_day_-1 | 66 | 36 | 0.000 | 0.005 |
| Baby_1_day_1 | Baby_2_day_14 | 66 | 43 | 0.007 | 0.069 |
| Baby_1_day_1 | Baby_2_day_28 | 66 | 42 | 0.005 | 0.054 |
| Baby_1_day_28 | Baby_2_day_1 | 73 | 68 | 0.633 | 1.000 |
| Baby_1_day_28 | Baby_2_day_-1 | 73 | 36 | 0.000 | 0.000 |
| Baby_1_day_28 | Baby_2_day_14 | 73 | 43 | 0.000 | 0.007 |
| Baby_1_day_14 | Baby_2_day_-1 | 99 | 36 | 0.000 | 0.000 |
| Baby_1_day_14 | Baby_2_day_28 | 99 | 42 | 0.000 | 0.000 |
| Baby_1_day_14 | Baby_2_day_1 | 99 | 68 | 0.000 | 0.005 |

**Table S4. Gastrointestinal pathogens to be screened in donor feces using the** BioFire kit

| **BACTERIA** | - 1. *Campylobacter (C. jejuni / C. coli / C. upsaliensis)*   2. *Clostridioides (Clostridium) difficile*(toxin A/B)   3. *Plesiomonas shigelloides*   4. *Salmonella*   5. *Yersinia enterocolitica*   6. *Vibrio (V. parahaemolyticus / V. vulnificus / V. cholerae)*   7. *Enteroaggregative E. coli*(EAEC)   8. *Enteropathogenic E. coli*(EPEC)   9. *Enterotoxigenic E. coli*(ETEC)*lt/st*   10. *Shiga-like toxin-producing E. coli*(STEC) *stx1/stx2*   11. *E. coli*O157   12. *Shigella/Enteroinvasive E. coli*(EIEC) |
| --- | --- |
| **VIRUSES** | 1. Adenovirus F40/41 2. Astrovirus 3. Norovirus GI/GII 4. Rotavirus A 5. Sapovirus (I, II, IV, and V) |
| **PARASITES** | 1. *Cryptosporidium* 2. *Cyclospora cayetanensis* 3. *Entamoeba histolytica* 4. *Giardia lamblia* |

**Table S5. Additional gastrointestinal pathogens to be screened in donor feces.**

| **Gastrointestinal pathogen screened** | **PCR** | **Culture** | **Microscopic examination** |
| --- | --- | --- | --- |
| ***Isospora*** | ✓ |  |  |
| ***Microsporidia*** | ✓ |  |  |
| ***Enterovirus*** | ✓ |  |  |
| **Methicillin Resistant *Staphylococcus aureus* (MRSA)** |  | ✓ |  |
| **Extended-spectrum β-lactamase (ESBL)–producing Enterobacteriaceae** |  | ✓ |  |
| **Vancomycin-Resistant *Enterococcus* (VRE)** |  | ✓ |  |
| **Carbapenem-resistant Enterobacteriaceae (CRE)** |  | ✓ |  |
| **Ova/Parasites** |  |  | ✓ |
